# Connectivity of Tiger (*Panthera tigris*) Populations in the Human-Influenced Forest Mosaic of Central India

**DOI:** 10.1371/journal.pone.0077980

**Published:** 2013-11-06

**Authors:** Aditya Joshi, Srinivas Vaidyanathan, Samrat Mondol, Advait Edgaonkar, Uma Ramakrishnan

**Affiliations:** 1 Post-graduate Program in Wildlife Biology and Conservation, Wildlife Conservation Society-India Program, National Centre for Biological Sciences, Tata Institute of Fundamental Research, Bangalore, India; 2 Foundation for Ecological Research, Advocacy & Learning, Pondicherry Campus, Auroville Post, Tamil Nadu, India; 3 National Centre for Biological Sciences, Tata Institute of Fundamental Research, Bangalore, India; 4 Indian Institute of Forest Management, Nehru nagar, Bhopal, India; Università degli Studi di Napoli Federico II, Italy

## Abstract

Today, most wild tigers live in small, isolated Protected Areas within human dominated landscapes in the Indian subcontinent. Future survival of tigers depends on increasing local population size, as well as maintaining connectivity between populations. While significant conservation effort has been invested in increasing tiger population size, few initiatives have focused on landscape-level connectivity and on understanding the effect different landscape elements have on maintaining connectivity. We combined individual-based genetic and landscape ecology approaches to address this issue in six protected areas with varying tiger densities and separation in the Central Indian tiger landscape. We non-invasively sampled 55 tigers from different protected areas within this landscape. Maximum-likelihood and Bayesian genetic assignment tests indicate long-range tiger dispersal (on the order of 650 km) between protected areas. Further geo-spatial analyses revealed that tiger connectivity was affected by landscape elements such as human settlements, road density and host-population tiger density, but not by distance between populations. Our results elucidate the importance of landscape and habitat viability outside and between protected areas and provide a quantitative approach to test functionality of tiger corridors. We suggest future management strategies aim to minimize urban expansion between protected areas to maximize tiger connectivity. Achieving this goal in the context of ongoing urbanization and need to sustain current economic growth exerts enormous pressure on the remaining tiger habitats and emerges as a big challenge to conserve wild tigers in the Indian subcontinent.

## Introduction

Habitat loss, prey depletion and poaching have severely affected wild tiger populations. Around 3,600 adult tigers occur in less than 7% of their historical range [1]. Despite drastic decline in their habitat and numbers, the Indian subcontinent remains the stronghold for long-term tiger persistence, and harbours nearly 60% of the global population of wild tigers [2]–[4]. Most of these individuals currently exist in small and isolated Protected Areas (PAs); which are too small to even hold demographically viable populations [3]. As a result, recent conservation strategies emphasize the need to expand conservation efforts to include more than one meta-population [5]. These efforts have led to identification of “Tiger Conservation Landscapes” (TCLs), which include a number of PAs interconnected by corridors that could potentially support viable populations [6].

The success of these meta-population/landscape conservation approaches depends critically upon detailed understanding of population dynamics, distribution and dispersal events. Earlier studies have shown that tiger abundance is driven by prey density [7], while tiger spatial distribution is driven by prey densities and human disturbance [8]. Successfully implementing a landscape-level conservation approach would require knowledge about tiger dispersal and factors influencing such dispersal events, which is lacking. However, very little is known about tiger dispersal rates, landscape predictors (e.g. roads, vegetation types, human habitation) and within PA conditions (e.g. prey density, tiger density) influencing such dispersal events. For large mammalian carnivores it is difficult to measure connectivity and dispersal, as long-distance dispersal events are rare [9]. Radio-telemetry [10]–[12] and camera trapping [13] can be used to study dispersal events. However, both these approaches require long-term monitoring across large landscapes, and conclusions can be made only if there are successful dispersal events during the study period.

Approaches involving genetic analyses provide a complementary tool to the above methods as they can provide information about population connectivity and relatively recent dispersal events. Advances in landscape genetics allow evaluation of the landscape effects on genetic structure and population connectivity [14], providing the ability to potentially test the impacts of specific mitigation measures on connectivity in the future. Genetic studies in northwest India indicates that tigers moved between two tiger reserves, which were 120 km apart [15], while that from central India suggest connectivity over distances of around 200 km [16]. These results come from Protected Areas that have relatively contiguous tiger habitat between them, or have potential corridors for movement exist, and do not highlight the factors influencing connectivity. Understanding elements of the landscape that facilitate connectivity and dispersal is crucial to successfully manage tiger populations at a landscape level.

In this study we combine genetic approaches with landscape ecology to study tiger dispersal between six PAs of the Central Indian landscape, and examine how the nature of the intervening landscape elements influences their dispersal. We define connectivity as a larger ecological measurement of gene flow from one population to another by immigrating individuals. The Central Indian landscape supports one of the largest tiger populations in India, and has been identified as a global tiger conservation landscape [5]. Its configuration (in terms of tiger population, number of PA’s, and mean distance between them) makes this landscape a potentially good model system to investigate landscape-level connectivity. However, over the last decade it has witnessed large-scale land use modification with increasing urbanization, expansion of highways, mining within and around forested habitats and increase in tourism. This has led to speculation that tiger populations within this landscape are isolated or are under threat of isolation, and recent efforts to mitigate the barrier effects of roads have gained importance at a national level [17].

We specifically ask the following questions: (1) Are tigers dispersing between populations in the Central Indian landscape, resulting in population connectivity? and (2) What landscape features, if any, affect connectivity in this landscape? We discuss the implications of our results for tiger conservation in human-dominated landscapes, with a particular focus on incorporating connectivity in the effective planning and management of existing PA networks and intervening areas.

## Methods

### Ethics

All field-based sampling was conducted non-invasively, without animal handling. Permissions to conduct research in the various Protected Areas were obtained from the relevant Forest Departments (Permit No./Technical-1/6473 and Permit No./D-22(8)/Research/3255/2009-10). Institutional biosafety and bioethics permissions were obtained from the National Centre for Biological Sciences, TIFR.

### Study Area

**Figure 1:**
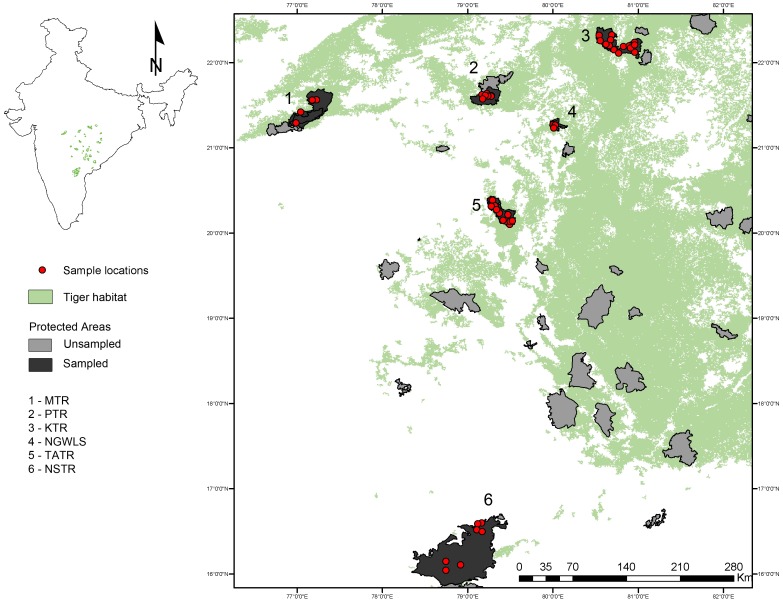
Study area. Map of the study area showing the tiger-habitat and sample locations.

The study was carried out in six PAs encompassing the major tiger populations of central India (Figure 1). These PAs are situated at varying distances from each other (Table S1) and spread across three states. Melghat Tiger Reserve (MTR), Pench Tiger Reserve (PTR), Nagzira Wildlife Sanctuary (NGWLS), and Tadoba-Andhari Tiger Reserve (TATR) are in the state of Maharashtra; Kanha Tiger Reserve (KTR), in the state of Madhya Pradesh and Nagarjunasagar- Srisailam Tiger Reserve (NSTR), in the state of Andhra Pradesh.

### Field Surveys

We obtained tiger DNA from faecal samples collected from the six PAs. Tigers are known to use roads and trails for travelling and regularly mark their territories by depositing faeces. Existing roads and trails in the PAs were searched for fresh tiger scats. Each road or trail was sampled only once to avoid recaptures and maximize the number of different individual in any of the area covered. Scat samples (n = 96) were stored in absolute ethanol and the geographical coordinates were recorded in field. The data collected in this study were combined with data from 22 other individual tigers (15 obtained from Mondol et al., [4] sampled in 2008 and seven from the Centre for Cellular and Molecular Biology, India sampled in 2010).

### Laboratory Methods

DNA was extracted from scat samples using QIAamp DNA stool mini kit (QIAGEN Inc.) as described in Mondol et al., [18]. Species identification was conducted using tiger-specific primers [19]. Based on expected heterozygosity, polymorphism and amplification success rates from previous studies in the laboratory, 14 microsatellite loci (FCA069, FCA090, FCA126, FCA230, FCA232, FCA279, FCA304, FCA441, FCA628, FCA672, [4], [18], [20], [21] MSHDZ170, MSFCA453, MSF115 and MSFCA506 [22] were used. PCR products were multiplexed and run with LIZ 500 size standard in an automated sequencer ABI3100XL (Applied Biosystems). Alleles were visualized using GENEMAPPER version 4.0 (Applied Biosystems) and scored manually.

### Data Quality

To minimize genotyping errors, amplification and scoring of each locus was repeated four times (for each sample) and a quality index (as in Mondol et al., [18]) was calculated. Only samples with 0.75 or higher quality were retained for further analyses. Program MICROCHECKER 2.2.3 [23] was used to estimate null alleles and scoring errors due to stutter peaks on the complete dataset.

### Population Genetic Analyses

#### Individual identification

Unique individuals (based on data from 12 or more loci) were identified using the program CERVUS [24]. Program GIMLET [25] was used to calculate Probability of Identity for siblings (P_ID-sibs_) for the loci used.

#### Genetic diversity

Genetic diversity in terms of alleles per locus (Al), expected heterozygosity (H_E_) and observed heterozygosity (H_0_) and Hardy Weinberg equilibrium were calculated using GENEPOP [26]. Pairwise F_ST_ was estimated between protected areas using program ARLEQUIN 3.1 [27]. Since F_ST_ represents connectivity over the last 150 to 200 years [28], recent connectivity was assessed using the proportion of shared alleles (D_SP_, see Landguth et al., [28] for justification), calculated using the program MICROSAT [29] and immigration/emigration rates (estimated using BayesAss 3 [30], see for Munshi-South [31] for justification).

#### Relatedness

We used program ML-RELATE [32] to calculate maximum likelihood estimates of pair-wise relatedness and relationship categories between individuals from genotypic data. Allele frequencies, pair-wise genetic relatedness, and kinship category estimations were performed by entering all individuals’ genotype as a single population, as well as separate populations (sampling populations) during analyses.

#### Population structure

We used a Bayesian clustering approach implemented in program STRUCTURE 2.3.2 [33], [34] to identify population structure in our data set. We evaluated the most likely number of clusters (K), testing values between one and ten, using one million iterations and a burn-in of 500,000, assuming correlated allele frequencies. We repeated the analyses ten independent times and finally the optimal value of K was calculated using STRUCTURE HARVESTER web version [35].

#### Detection of migrants

To detect tiger dispersals in this landscape, we used three different approaches that use allele frequencies to detect migrant individuals in our dataset. First, we used prior population information in the USEPOPINFO option implemented in STRUCTURE 2.3.2 to detect first-generation migrants in our sampled populations. Run conditions used for this analysis were as described above. We assigned different migration rates (MIGPRIOR 0.01, 0.02, 0.05, 0.1) as a sensitivity test during the analysis. We ran the analysis with two different groups of data: a) individuals grouped as populations according to their sampling locations and b) individuals grouped as genetic clusters from our initial run. These different runs helped us to check consistency of the results across different genetic groups created.

Further, we used ‘Migrant detection’ function in program GENECLASS 2.0 [36] to identify first generation migrants. We used a Bayesian approach as described by Rannala and Mountain [37] along with the resampling method of Paetkau et al., [38] for likelihood computation (L_home/L_max), with 10000 simulated individuals at an assignment threshold (alpha) of 0.01. This method allows detection of migrants even when the overall differentiation between populations is low. Apart from first generation migrant detection, we did individual assignments in GENECLASS using Bayesian criterion of Rannala and Mountain [37] in combination without resampling with rest of the parameters same as described above.

Finally, we used a Bayesian assignment approach implemented in program SCAT ver. 2.0 [39], [40] to support our GENECLASS and STRUCTURE results. We assigned all individuals found as migrants (as indicated by both previous analyses) to this landscape against reference samples (found as resident individuals during our previous analyses). The advantage of this approach lies in its use of geographic location information from the reference samples, and resulting assignment of each unknown individual to a geographic location. SCAT uses allele frequencies from reference samples and spatial smoothing methods [41] to generate a geographic map of allele frequency variation across the entire sampling area (including intervening areas without reference samples). Reference allele frequencies were generated based on the Smoothed Continuous Assignment Technique, using a Markov Chain Monte Carlo (MCMC) scheme [39], [40]. Observed alleles from the dispersed tigers were then compared with the geographic map of tiger allele frequencies from that sample’s respective habitat to determine the range of plausible locations for each sample. We initially conducted exploratory runs with multiple combinations of input parameters (burn-in, thinning and iterations) with the entire data to select the best parameter combinations. After comparing the results across test runs, ten independent runs with lengths of 100 burn-ins, 10 thinning and 100 iterations were performed. All resulting likelihood values were compiled excluding the initial burn-in data. We then estimated the 99% and 95% kernel density of assignment to identify the uncertainty associated with sample assignment. Final plots included the original sampling location, the median latitude and median longitude of assignment, and the kernel density (both 99% and 95%, representing uncertainty as a contour) on an India map.

### Landscape-resistance Models

Radio-collaring studies on tigers indicate maximum 30 km dispersal distance for adult males [10]. While this may not extend to all habitats, it is the only information available on direct dispersal; hence we used this as a maximum dispersal distance to delineate our study landscape. We buffered all forested patches up to a distance of 30 km to define the study landscape. Available literature reveals that tigers are able to disperse through a wide range of forested habitats, occasionally through agricultural land but avoiding urbanized areas [10]. Consequently, we masked out all urban areas to further refine the study landscape. Night-lights [42], [43]were used to identify urbanized areas; all pixels with values >20 were categorized as urban centres as these represented class I–IV towns with populations more than 10,000 people (data obtained from Census of India 2001). To identify landscape features that differentially influence dispersal and gene flow, landscape resistance layers based on proximity between tiger habitat, percentage tree cover, proximity between human settlements and road densities were developed. Each of these layers has different spatial resolution, we used the best possible resolution and all layers were re-sampled to a spatial resolution of 1 km^2^. To assess the relative influence of various landscapes elements, we rescaled all resistance layers to range from 1 to 100.

#### Tiger habitat

We used the MODIS land cover maps (MCD12Q1), IGBP global vegetation classification scheme to identify potential tiger habitats. The original data has 17 land-cover classes at a resolution of 500 m and was generated following the algorithm described by Friedl et al., [44] using training datasets. We are aware that limited number of training sites could have been used to generate these global land-cover maps. Using a layer of forest administrative boundaries we identified major classes that represented forested habitats in Central India. These include all forest types and savannas, we combined all such patches >10 km^2^ [6] to represent potential tiger habitats and assigned a minimum resistance value of 1. The Euclidean distance between patches of tiger habitat, a measure of proximity between patches of tiger habitat was used to assign landscape resistance to dispersal from one tiger habitat to another; larger distances represented higher resistances.

#### Tree cover

We used remotely sensed MODIS Vegetation Continuous Fields data (MOD44B) to measure percentage tree cover across the landscape for the year 2009. The original 250 meters dataset provides a gradation of percent tree cover across the landscape and ranged between 0–67 percent, this was rescaled to 1–100 to obtain a resistance layer where pixels with lower tree cover were assigned higher resistance values.

#### Human settlements

Remotely sensed nightlights data reflect human densities/urbanization [42], [43]. Radiance calibrated average night-light data at 1 km resolution for 2009 was obtained from the National Geophysical Data Centre (available at http://www.ngdc.noaa.gov/dmsp/download_rad_cal_96-97.html). All illuminated pixels were extracted to derive a layer of human-settlements. We are aware that this would be a subset of all settlements within the landscape as a large number of smaller settlements without electrification will not be identified. Again, Euclidean distance from the edge of the settlement was used to assign resistance to dispersal; larger distances represented lower resistances and shorter distances or areas in close proximity to human settlements were assigned higher resistance.

#### Roads

Using a vector layer of national highways, state highways and major public roads we generated a raster layer of road densities at a resolution of 1 km. Areas with higher road densities were assigned higher resistance values and those without any road network were assigned the minimum resistance value of 1.

### Recent Connectivity and Landscape Resistance

A pair-wise algorithm in Circuitscape 3.5.7 (based on circuit theory) was used to estimate landscape resistance between sampled sites [45]. While the pair wise algorithm assumes a uniform source strength of 1 each of sampled region (polygon), we used the corresponding tiger numbers (number of camera trapped individuals) [2] as source strength in our calculations. We used number of camera-trapped individuals as a surrogate for tiger densities as they might be less biased than the density estimates in Jhala et al., [2] are based on limited sampling [46]. A non-parametric Mantel test (where significance is assessed by permutations) was used to assess whether pair-wise differences in genetic differentiation (D_SP_/immigration/emigration rates) between sampled sites correlated with pairwise differences in landscape resistances. Mantel tests were implemented in R [47] using package ecodist [48]. We also investigated whether F_ST_ (historical connectivity) is affected by geographic distance. In order to maintain consistency, we used the same Mantel tests assuming that resistance was only caused by Euclidean distance.

## Results

### Data Quality and Genetic Diversity

From all 96 field-collected faecal samples, we identified 48 tigers (50%) and 10 leopards (10%). Remaining 38 samples (39.5%) were unresolved. Using a cut-off criterion of 12 or more loci data, we identified 33 unique tigers from these samples, without any recaptures. Our final data comprised a total of 55 individuals (22 additional individuals described in methods section). These data represent about 30% of the estimated tiger population in this entire landscape [2]. Numbers of individual tigers from different PAs were as follows: MTR: 5, PTR: 7, NGWLS: 5, TATR: 16, KTR: 15 and NSTR: 7.

None of the 14 loci showed deviations from Hardy-Weinberg Equilibrium (HWE), while only one locus (msFCA506) showed presence of null alleles. All the loci were polymorphic with a mean of 11.71 alleles (ranging from 5 to 16 alleles), expected heterozygosity (HE) of 0.81 and observed heterozygosity (Ho) of 0.54 (Table S2).

Overall, we found low average relatedness value (0.054) and very few related individuals in the dataset. The relatedness analyses show only seven pairs of individuals with relatedness value higher than 0.5. These include individuals mostly from Tadoba and Nagzira. None of these individuals are assigned a ‘migrant’ status by any of our assignment approaches. The overall relatedness among individuals is presented in figure S1.

### Population Structure and Genetic Differentiation

**Figure 2:**
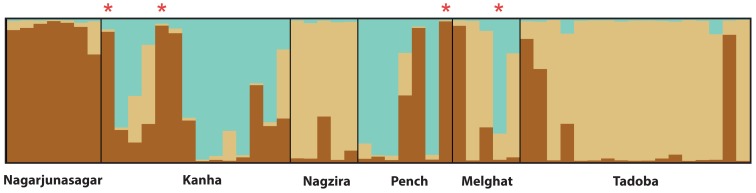
Population structure in the Central Indian landscape. STRUCTURE plot showing the distribution of genetic variation (at K = 3). Individuals with ‘*’ mark indicate migrants detected by STRUCTURE.

The STRUCTURE analysis indicates presence of three distinct genetic clusters (K = 3, based on ten independent runs) in this landscape (Figure 2). Our data reveal that tiger populations are genetically differentiated at low, but significant levels (Table 1) for almost all populations. We used D_SP_ as an indicator of relatively recent connectivity. Similar to F_ST_, D_SP_ values are relatively low (high values could be as large as 9 or 10), indicating that populations share alleles. Like with F_ST_, we can be reasonably confident of our D_SP_ estimates, as standard errors were less than 10% (Table 1). Finally, both emigration and immigration rates (Table S3, S4) are relatively low, suggesting a relatively low recent connectivity.

**Table 1:** F_ST_ (above diagonal, genetic differentiation, historical connectivity, significant values in bold) and D_SP_ (below diagonal, genetic similarity, recent connectivity, standard error in parentheses).

	Pench	Melghat	Tadoba	Nagzira	Kanha	Nagarjun- sagar
Pench		**0.0907**	**0.1438**	**0.2167**	0.0564	**0.0735**
Melghat	0.937 (0.13)		**0.126**	**0.1679**	**0.0958**	**0.084**
Tadoba	1.083 (0.12)	0.773 (0.12)		**0.1035**	**0.1605**	**0.1462**
Nagzira	1.172 (0.15)	0.94 (0.11)	0.762 (0.14)		**0.1789**	**0.1461**
Kanha	0.789 (0.11)	0.829 (0.09)	0.85 (0.11)	1.006 (0.14)		0.0321
Nagarjun-sagar	1.11 (0,11)	1.017 (0.15)	0.986 (0.14)	1.156 (0.09)	0.899 (0.08)	

### Connected Tiger Populations in the Central Indian Landscape

Migrant detection in STRUCTURE identified four individuals with migrant probability >0.9 at K = 3 (Table 2). The inferred ancestry of individuals did not vary across different assumed values of migration rate (MIGPRIOR of 0.02, 0.05 and 0.1). Out of these four migrants, two individuals from KTR were assigned to NSTR, one from PTR to NSTR and one from MTR to KTR.

**Table 2:** Results of migrant detection analyses. *p resident* refers to the probability of an individual belonging to the population from where it was sampled.

Tiger ID	Sampled in	Assigned Population	GeneClass *F_0_* Log ratio(L_home/L_max)	*p* Resident	STRUCTURE migrant probability (MIGPRIOR = 0.05)
**NSTR6**	NSTR	KTR	1.633	0.006	0.002
**NSTR7**	NSTR	NGWLS	2.562	0.004	0.053
**KNHR2**	KTR	NSTR	4.523	0.002	**0.977**
**KNHR6**	KTR	NSTR	5.116	0.001	**0.984**
**KNH13**	KTR	MTR	1.611	0.008	0.001
**KNH15**	KTR	MTR	2.333	0.004	0.012
**PNCR7**	PTR	KTR	3.921	0.001	0.195
**PNCR8**	PTR	NSTR	4.05	0	**0.9409**
**MELR1**	MTR	NGWLS	3.206	0.003	0
**MELR4**	MTR	KTR	5.429	0.001	**0.913**

(NSTR is Nagarjunasagar, KTR is Kanha, PTR is Pench, NGWLS is Nagzira and MTR is Melghat).

**Figure 3:**
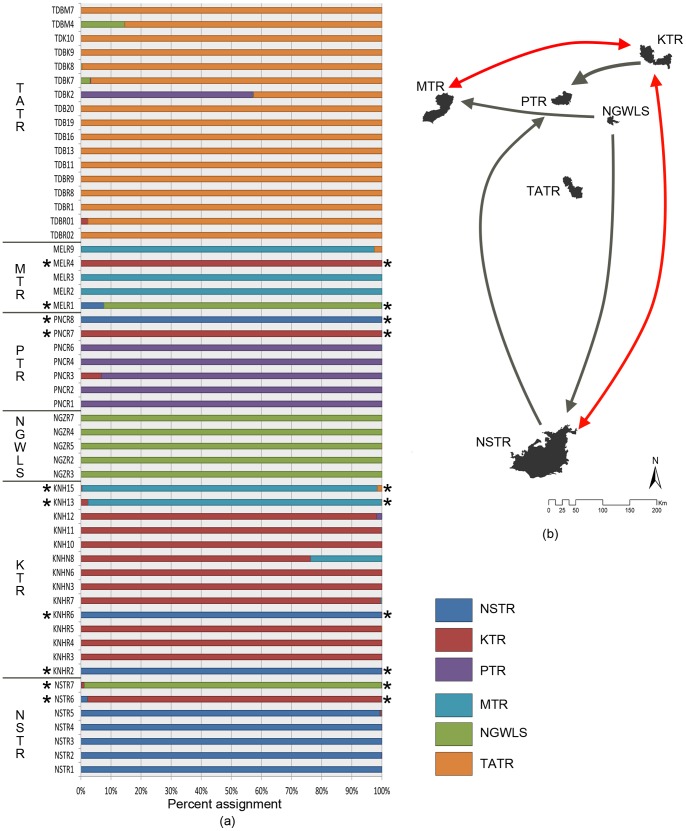
Individual assignment across populations. (a) Barplot showing the percent assignment of each individual to a population. Each bar represents an individual and the colour represents the population to which it was assigned. The highlighted bars (*) represent the dispersed individuals. Score of >95% was used as a threshold for complete assignment (b) the inferred Tiger dispersal events over varying distances. The assignment results suggest that apart from relatively short distance (i.e. ∼165 km, between KTR & PTR) individuals are also moving over very large distances, on the order of 600 km (NSTR-KTR and PTR-NSTR), 500 km (NSTR-NGWLS) and 300 km (MTR-KTR and MTR-NGWLS).

In addition to these four migrants detected by STRUCTURE, GENECLASS identified six additional first generation (*F_o_*) migrants (Table 2). Results for first-generation migrant detection and assignment without resampling were the same (Table 2 and Figure 3). In both cases, a total of ten individuals were assigned to areas different from their sampled locations. The results show connectivity between NSTR-KTR, PTR-NSTR, NSTR-NGWLS, KTR-PTR, KTR-MTR and MTR-NGWLS. However, TATR seems to be poorly connected, as there are no immigrants or emigrants detected from this population.

**Figure 4:**
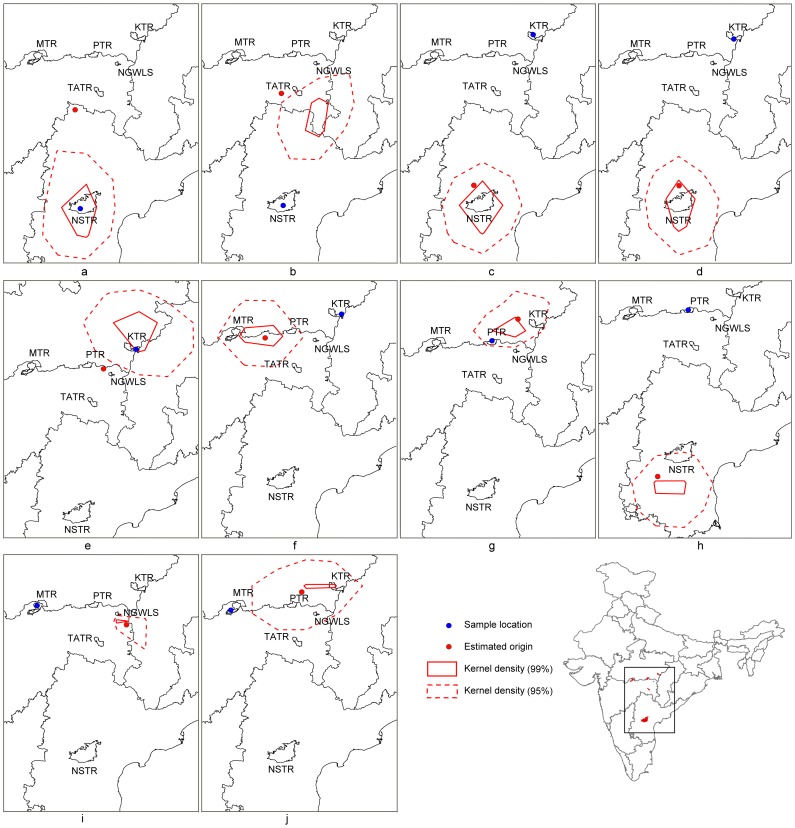
SCAT plots showing uncertainty in tiger assignment. The blue-coloured dot is the original sampling location; the red-coloured dot is the median point of assignment. The uncertainty is represented by the 99% & 95% contour. **a (NSTR6), b (NSTR7) are** individuals sampled in NSTR; **c (KNHR2), d (KNHR6), e (KNH13), f (KNH15) are individuals** sampled in KTR; **g (PNCR7), h (PNCR8)** are individuals sampled in PTR and **I (MELR1), j (MELR4)** are individuals sampled in MTR.

Results from additional analyses conducted in SCAT, on dispersed individuals identified by GENECLASS and STRUCTURE re-iterate our inference of long-distance dispersal. Figure 4 shows median assignment of individuals to geographic location along with the associated uncertainty. Apart from three sample (NSTR6, NSTR7 and KTR13), all individuals are assigned very close to the PAs suggested by the GENECLASS and STRUCTURE analysis.

### Landscape affects Population Connectivity across the Central Indian Landscape

Neither F_ST_ nor D_SP_/immigration/emigration rates were significantly correlated with Euclidean distance (Table 3), suggesting absence of Isolation by Distance (IBD). Mantel tests revealed that recent connectivity (D_SP_) was correlated with the presence of human settlements (r^2^ = 0.21, p = 0.033, Table 4), and combinations of landscape elements that included human settlements, roads, tree cover and tiger habitat (Table 4). Emigration rate (movement from a protected area) was significantly impacted by tiger habitat (Table 5, r^2^ = 0.11, p = 0.043), roads and tiger habitat (r^2^ = 0.11, p = 0.049) and human settlements and tiger habitat (Table 5, r^2^ = 0.10, p = 0.051). Immigration rate was not significantly related to any landscape elements or combinations thereof (Table 6). Figure S2 depicts landscape connectivity based on combined resistance from human settlements, roads and forest cover in the Central Indian landscape.

**Table 3:** Mantel tests between various measures of genetic subdivision and geographic distance.

Statistic	r^2^	p value
F_ST_	0.0435	0.42
D_SP_	0.107	0.53
Immigration rate	0.007	0.79
Emigration rate	0.104	0.29

**Table 4:** Non-parametric partial Mantel tests between D_SP_ and landscape resistance (significant values at p = 0.05 level in bold).

	r^2^	p
**Human settlements**	**0.206**	**0.033**
**Human settlements, Roads, Tree cover**	**0.205**	**0.035**
**Human settlements, Roads**	**0.205**	**0.036**
**Human settlements, Tiger habitat**	**0.203**	**0.038**
**Human settlements, Roads, Tiger habitat**	**0.203**	**0.043**
**Human settlements, Tree cover**	**0.201**	**0.044**
**Human settlements, Tree cover, Tiger habitat**	**0.199**	**0.049**
Human settlements, Tree cover, Tiger habitat, Roads	0.199	0.053
Tiger Habitat	0.196	0.053
Roads, Tiger habitat	0.194	0.067
Tree cover, Tiger habitat	0.192	0.071
Roads, Tree cover, Tiger habitat	0.192	0.071
Tree cover	0.193	0.073
Roads+Tree cover	0.193	0.073
Roads	0.188	0.098

**Table 5:** Non-parametric partial Mantel tests between emigration rate and landscape resistance (significant values at p = 0.05 level in bold).

	r^2^	p
**Tiger Habitat**	**0.109**	**0.043**
**Roads, Tiger habitat**	**0.11**	**0.049**
**Human settlements, Tiger habitat**	**0.107**	**0.051**
Human settlements	0.106	0.053
Human settlements, Roads, Tiger habitat	0.106	0.054
Human settlements, Roads	0.106	0.056
Roads	0.109	0.057
Human settlements, Roads, Tiger habitat, Roads	0.106	0.062
Human settlements, Tree cover	0.105	0.063
Roads, Tree cover, Tiger habitat	0.106	0.064
Human settlements, Tree cover, Tiger habitat	0.106	0.064
Human settlements, Roads, Tree cover	0.106	0.065
Tree cover, Tiger habitat	0.105	0.068
Tree cover	0.104	0.071
Roads, Tree cover	0.106	0.071

**Table 6:** Non-parametric Mantel tests between immigration rate and landscape resistance.

	r^2^	P
Human settlements, Tiger habitat	0.0123	0.389
Human settlements, Road	0.0123	0.389
Human settlements, Tree cover, Tiger habitat	0.0117	0.39
Human settlements, Roads, Tree cover	0.0114	0.39
Human settlements, Tree cover, Tiger habitat, Roads	0.0114	0.391
Human settlements, Roads, Tiger habitat	0.0119	0.391
Tree cover, Tiger habitat	0.0104	0.393
Human settlements, Tree cover	0.0119	0.393
Tree cover	0.0079	0.395
Roads, Tree cover, Tiger habitat	0.01	0.398
Tiger Habitat	0.0098	0.399
Roads, Tiger habitat	0.0098	0.4
Human settlements	0.0102	0.402
Roads, Tree cover	0.0096	0.405
Roads	0.0121	0.407

## Discussion

### Tigers are Dispersing much Greater Distances than Previously Found

Assignment tests, historical patterns of population connectivity and measures of recent geneflow indicate population connectivity in the Central Indian landscape through seemingly tiger-inhospitable habitats. This is in concordance with the results from a recent study done in the Satpura-Maikal (a portion of the Central Indian landscape) area [16], which suggested low population structure, implicating connectivity.

Genetic assignment tests identified individuals that migrated within the last generation; our results support long-distance dispersal by individual tigers of over 690 km, suggesting that either this individual (*F_o_* migrant) or its parents moved this distance. The assignment tests performed in SCAT reinforce our results, with high certainty in these estimates. The traditional problem with assignment studies is the inability to assign uncertainty to these assignments. Our use of SCAT in this context is very powerful, and could be henceforth added to the suite of landscape genetic analyses.

Even from a conservative perspective, this suggests a minimum dispersal distance of 345 km (if the parent moved half the distance, and the offspring, captured as a migrant in our data, moved the remaining distance). This is much higher than empirical dispersal distances for tigers suggested by camera trap studies (200 km [13]) and genetic data (200 km [15], [16]), and close to predicted median dispersal distances based on theoretical models that use allometric data (∼450 km [49]). While this extends the range of tiger dispersal two- to three-fold, it could still be an underestimate, given that radio-collared mountain lions, and other large carnivores, have been shown to disperse even longer distances [50]–[52].

One possible concern with our study is the relatively low sample size (55 individuals) compared to those in Sharma et al., [16] and its possible impact on the assignment results. Paetkau et al. [38] suggest that the Monte Carlo resampling methods such as implemented in Rannala and Mountain [37] are not ideal when trying to identify immigrants from a limited data set, leading to an increased inference of immigrants. The discrepancy we find between STRUCTURE and GENECLASS support their suggestions. GENECLASS detects a total of ten dispersal events, while STRUCTURE detects only four. Finally, SCAT appears to be an intermediate approach between these two, re-enforcing the individuals detected by STRUCTURE as migrants, but also suggesting two additional migrants (a total of six migrants). The fact that all approaches label the same individuals as migrants, and that even under the most conservative assumptions (STRUCTURE results) we find evidence for long-distance dispersal suggests that our inferences are robust to assignment methods and their varying assumptions.

Despite lower sample size, our F_ST_ values are not qualitatively different from those observed for tigers in these and other populations [15]. Additionally, it appears that our results provide better power to detect population subdivision (compared to [16]). This is possibly because we use a large number of markers, which results in greater power to detect population subdivision. Simulation studies investigating trade-offs between sample size and number of loci have suggested similar patterns for landscape genetic processes [53].

Our results show that dispersal is occurring not just across long distances, but also across a highly fragmented and human-dominated landscape. Mapping current flow allows us to visualize how such connectivity may be achieved (Figure S2). While our study does not aim to identify corridors for movement, resistance based approaches are useful in identifying landscape elements that limit gene flow in the Central Indian tiger population. The burgeoning field of movement and dispersal ecology, which addresses behavioural decisions made by animals with respect to dispersal, as well as the habitats selected for such events, will provide a framework to further investigate such phenomena.

### Direct Human Footprint Affects Connectivity

The presence of human settlements has a negative impact on tiger connectivity (Table 4). Apart from this single parameter, all Mantel tests that are significant include human settlements along with other landscape variables. D_SP_ provides more explanatory power compared to migration rate estimates (higher r^2^ values, Tables 5 and 6). When significant, in addition to human footprint (roads and human settlements), tiger habitat also has an impact on connectivity. Several studies have shown that roads form linear barriers to dispersing carnivores [54]–[56]. However, our results are amongst the first to show that human population density could be detrimental to connectivity [57]–[60]. Most studies addressing connectivity have been in temperate locations with developed economies, where human population density is low, unlike in tropical locations where the economy is developing and human population density is tenfold higher. We suggest that carnivores such as tigers might avoid locations of very high human population density, thus an increase in the direct human footprint (e.g. high density urban settlements) might be detrimental to connectivity.

### Other Factors Influencing Connectivity

Assignment results show that 70% of the dispersers are from/going to Kanha, the PA with the highest prey and tiger density in the study area. Our results support predictions (e.g. Walston et al., [1]) that populations with high density will participate more actively in maintaining connectivity. That dispersal depends positively on population density has been illustrated in several species [61]–[63]. Our results also suggest that low-density populations can have high migration rates (e.g. Nagarjunsagar Srisailam Tiger Reserve), suggesting that tigers may disperse out of sites with low prey abundances towards high quality habitats. Negative relationship between dispersal and density has been observed in some species (e.g. brown bears, [64]). Finally, certain populations appear isolated in spite of having a high density and structural connectivity (e.g. Tadoba-Andhari Tiger Reserve); although, it is possible that, TATR might have connectivity with other PAs that were not sampled in this study.

Apart from population density, population growth rates could also impact tiger dispersal. Detailed camera trap studies also provide information of demographic rates [65], and such information could be incorporated into future studies on connectivity.

### Implications for Tiger Conservation: Moving Beyond Populations

In discussing conservation strategies for tigers, Walston et al., [1] emphasize the need for well-managed protected areas, and locally increasing tiger population sizes. Wikramanayake et al., [5], [66], on the other hand, argue that a meta-population/landscape-based conservation strategy is required. Our findings indicate that meta-populations might already exist within current TCLs. To maintain and enhance existing connectivity between tiger populations, management strategies must address demographic viability (tiger numbers), but more importantly landscape and habitat viability outside and between PAs.

Human demographic studies predict that much of the world will be urbanized within the next century [67], and in India, studies have shown considerable increase in the number of new towns or urbanised areas over the last decade [68]. Additional studies in the tropics indicate detrimental land-use change around PAs [69]–[71]. Our results suggest that such unplanned development will greatly compromise landscape-level connectivity for tiger populations in Central India. Recent directives passed by the Supreme Court of India on buffer zones around tiger reserves to be identified and officially notified, makes our results relevant to on-going conservation efforts.

While these are broad generalities, our results (Figure 3, 4) re-establish the functionality of the Kanha-Pench corridor [as suggested by Sharma et al., [16]], where expansion of a national highway is currently being proposed. With more than 13% of sampled individuals dispersing within the landscape in the last decade, any “developmental activity” could hamper connectivity at a large scale. For the future, we need to identify and legally notify areas as critical wildlife corridors.

Given the increasing trend towards urbanization, how can we maintain connectivity? At the landscape scale, most corridors support local people, and future conservation strategies must include plans to provide earnings to communities based on forests conserved by them. Plans like Payments for Ecosystem Services (PES), REDD+ and certification based on wildlife presence will help in maintaining physical as well as functional connectivity in the landscape.

Around 3,000 tigers remain in the wild globally, and currently the focus of conservation is limited to increasing tiger populations. Our results reveal that some PAs can remain isolated despite high local population density. Increasing local tiger abundance, while important, will still be an inadequate conservation strategy in the absence of population connectivity. If dispersal translates into migration along with increases in tiger population size, the future of tigers is promising, provided that connectivity is maintained and becomes an important conservation agenda in the future.

## Supporting Information

Figure S1 **Relatedness between individuals.** A heatmap showing relatedness values between all individual in our dataset. Relatedness is presented in a continuous scale between 0 and 1, with values below 0.5 going from light brown to light green, while values between 0.5 and 1 go from light green to dark blue.
(TIFF)

Figure S2 **Landscape connectivity.** A map showing landscape connectivity based on the current output from *Circuitscape.*
(TIF)

Table S1 **Euclidean distance (in Km) between Protected Areas.**
(DOCX)

Table S2 **Genetic diversity and genotyping error details of 14 microsatellite loci used in this study.**
(DOCX)

Table S3 **Recent Emigration rates estimated using BayesAss.**
(DOCX)

Table S4 **Recent Immigration rates estimated using BayesAss.**
(DOCX)
