# Supplementary figures and images for: Connectivity of Tiger (Panthera tigris) Populations in the Human-Influenced Forest Mosaic of Central India
Source: PLoS One. 2013 Nov 6;8(11):e77980. doi: 10.1371/journal.pone.0077980 (PMC3819329; doi:10.1371/journal.pone.0077980)

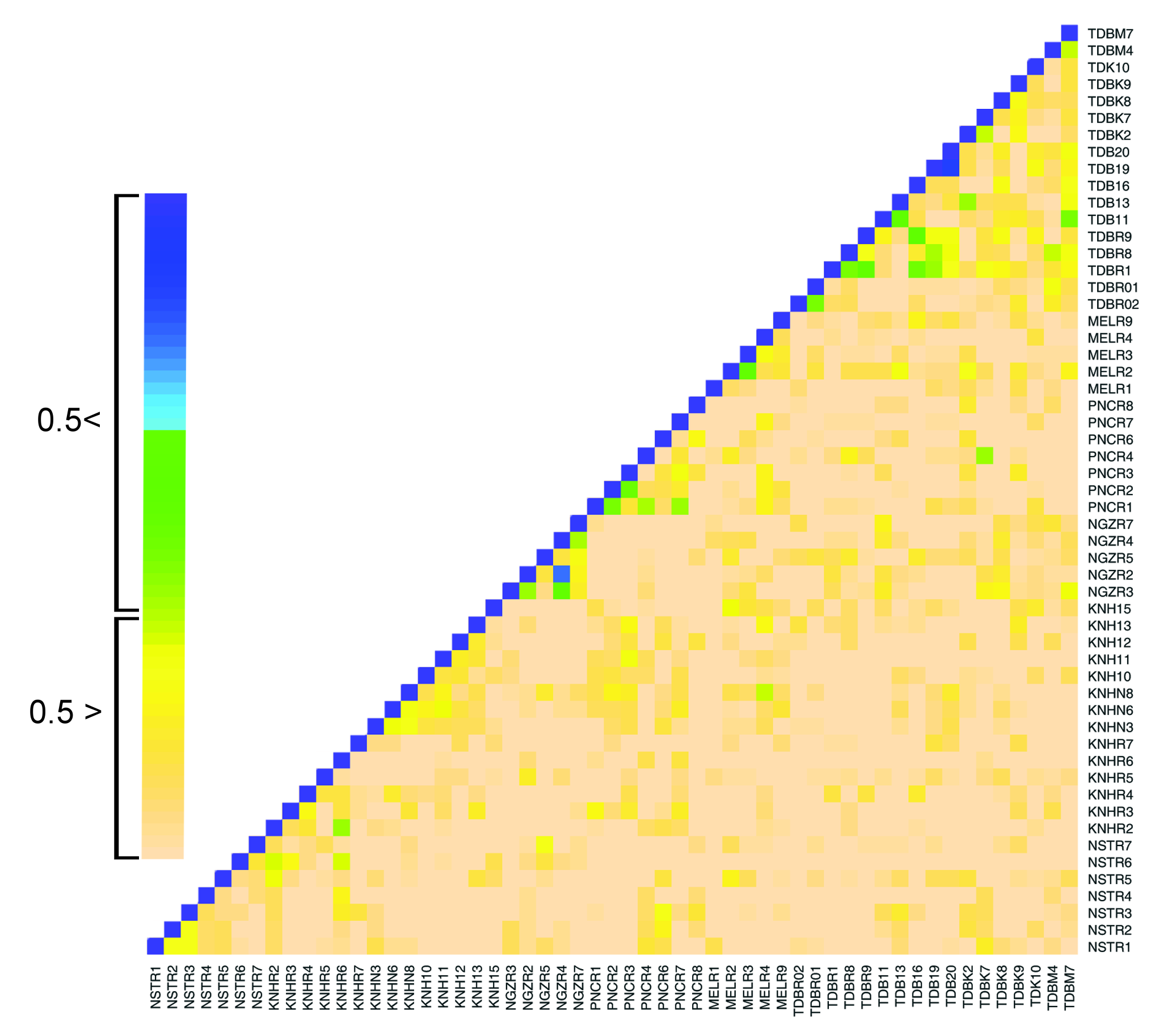

Supplement: Figure S1 — Relatedness between individuals. A heatmap showing relatedness values between all individual in our dataset. Relatedness is presented in a continuous scale between 0 and 1, with values below 0.5 going from light brown to light green, while values between 0.5 and 1 go from light green to dark blue. (TIFF) [file pone.0077980.s001.tiff]

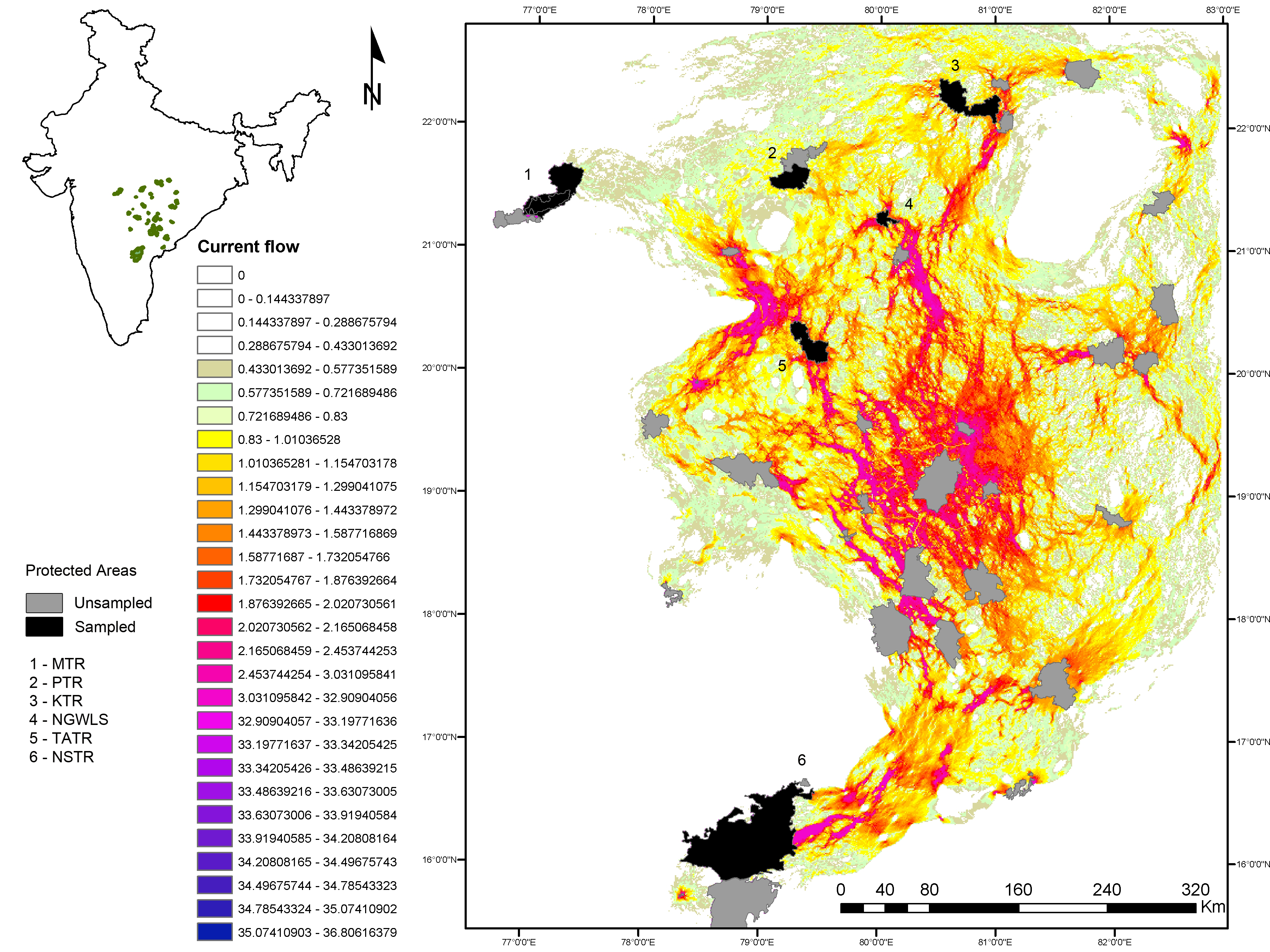

Supplement: Figure S2 — Landscape connectivity. A map showing landscape connectivity based on the current output from Circuitscape. (TIF) [file pone.0077980.s002.tif]
