# Supplementary material for: Connectivity of Tiger (Panthera tigris) Populations in the Human-Influenced Forest Mosaic of Central India
Source: PLoS One. 2013 Nov 6;8(11):e77980. doi: 10.1371/journal.pone.0077980 (PMC3819329; doi:10.1371/journal.pone.0077980)
Supplement: Table S1 — Euclidean distance (in Km) between Protected Areas. (DOCX) [file pone.0077980.s003.docx]

**Table S1: Euclidean distance (in Km) between Protected Areas**

|  | **Pench** | **Melghat** | **Tadoba** | **Nagzira** | **Kanha** |
| --- | --- | --- | --- | --- | --- |
| **Melghat** | 217.4 |  |  |  |  |
| **Tadoba** | 158.5 | 267.2 |  |  |  |
| **Nagzira** | 92.2 | 297.8 | 131.3 |  |  |
| **Kanha** | 165.6 | 379.3 | 260.7 | 129.5 |  |
| **Nagarjunsagar** | 605.8 | 610.1 | 449.6 | 572.0 | 693.4 |
