# Supplementary material for: Connectivity of Tiger (Panthera tigris) Populations in the Human-Influenced Forest Mosaic of Central India
Source: PLoS One. 2013 Nov 6;8(11):e77980. doi: 10.1371/journal.pone.0077980 (PMC3819329; doi:10.1371/journal.pone.0077980)
Supplement: Table S2 — Genetic diversity and genotyping error details of 14 microsatellite loci used in this study. (DOCX) [file pone.0077980.s004.docx]

**Table S2: Genetic diversity and genotyping error details of 14 microsatellite loci used in this study**

| **Locus** | **Repeat length** | **No. of alleles** | **H_E_** | **H_O_** | **Null allele** | **Allelic dropout (%)** | **False allele (%)** | **HWE** | **P_ID(sibs)_** | **Reference** |
| --- | --- | --- | --- | --- | --- | --- | --- | --- | --- | --- |
| FCA126 | 2 | 11 | 0.84 | 0.55 | No | 0 | 0.5 | Yes | 3.39 e^-1^ | Menotti-Raymond et al. (1999) |
| FCA069 | 2 | 13 | 0.77 | 0.57 | No | 0.5 | 1.5 | Yes | 1.30 e^-1^ | Menotti-Raymond et al. (1999) |
| FCA090 | 2 | 11 | 0.79 | 0.65 | No | 0 | 0 | Yes | 4.82 e^-2^ | Menotti-Raymond et al. (1999) |
| FCA304 | 2 | 16 | 0.86 | 0.61 | No | 0.5 | 1 | Yes | 1.58 e^-2^ | Menotti-Raymond et al. (1999) |
| FCA441 | 4 | 10 | 0.79 | 0.45 | No | 1 | 0.5 | Yes | 5.95 e^-3^ | Menotti-Raymond et al. (1999) |
| FCA672 | 2 | 11 | 0.83 | 0.59 | No | 0 | 0.4 | Yes | 2.07 e^-3^ | Menotti-Raymond et al. (1999) |
| FCA628 | 2 | 14 | 0.88 | 0.45 | No | 0.4 | 0.4 | Yes | 6.60 e^-4^ | Menotti-Raymond et al. (1999) |
| FCA232 | 2 | 12 | 0.72 | 0.57 | No | 0.4 | 0.9 | Yes | 2.75 e^-4^ | Menotti-Raymond et al. (1999) |
| FCA230 | 2 | 15 | 0.88 | 0.67 | No | 1 | 0 | Yes | 8.77 e^-5^ | Menotti-Raymond et al. (1999) |
| FCA279 | 2 | 13 | 0.84 | 0.6 | No | 0.4 | 0 | Yes | 2.96 e^-5^ | Menotti-Raymond et al. (1999) |
| msHDZ170 | 2 | 10 | 0.85 | 0.59 | No | 0 | 2.7 | Yes | 9.86 e^-6^ | Menotti-Raymond et al. (1999) |
| msFCA453 | 4 | 11 | 0.75 | 0.46 | No | 0 | 1.3 | Yes | 3.95 e^-6^ | Mondol et al. (2012) |
| msF115 | 4 | 5 | 0.67 | 0.49 | No | 0.4 | 0 | Yes | 1.81 e^-6^ | Mondol et al. (2012) |
| msFCA506 | 2 | 12 | 0.85 | 0.32 | Yes | 0 | 1 | Yes | 6.05 e^-7^ | Mondol et al. (2012) |
| **Mean** |  | **11.71** | **0.81** | **0.54** |  | **0.32** | **0.72** |  |  |  |
