# Supplementary material for: Connectivity of Tiger (Panthera tigris) Populations in the Human-Influenced Forest Mosaic of Central India
Source: PLoS One. 2013 Nov 6;8(11):e77980. doi: 10.1371/journal.pone.0077980 (PMC3819329; doi:10.1371/journal.pone.0077980)
Supplement: Table S3 — Recent Emigration rates estimated using BayesAss. (DOCX) [file pone.0077980.s005.docx]

**Table S3: Recent Emigration rates estimated using BayesAss**

| **Emigration (Bottom Matrix)** | | **From** | | | | | |
| --- | --- | --- | --- | --- | --- | --- | --- |
|  |  | **Pench** | **Melghat** | **Tadoba** | **Nagzira** | **Kanha** | **Nagarjunsagar** |
| **Into** | **Pench** | 0.822 |  |  |  |  |  |
|  | **Melghat** | 0.021 | 0.739 |  |  |  |  |
|  | **Tadoba** | 0.018 | 0.124 | 0.977 |  |  |  |
|  | **Nagzira** | 0.023 | 0.023 | 0.192 | 0.715 |  |  |
|  | **Kanha** | 0.073 | 0.009 | 0.040 | 0.009 | 0.840 |  |
|  | **Nagarjunsagar** | 0.012 | 0.013 | 0.019 | 0.014 | 0.038 | 0.903 |

Matrix shows the migration rates from the populations in vertical column to the population in the horizontal row
