# Supplementary material for: Connectivity of Tiger (Panthera tigris) Populations in the Human-Influenced Forest Mosaic of Central India
Source: PLoS One. 2013 Nov 6;8(11):e77980. doi: 10.1371/journal.pone.0077980 (PMC3819329; doi:10.1371/journal.pone.0077980)
Supplement: Table S4 — Recent Immigration rates estimated using BayesAss. (DOCX) [file pone.0077980.s006.docx]

**Table S4: Recent Immigration rates estimated using BayesAss**

| **Immigration (Top Matrix)** | | **From** | | | | | |
| --- | --- | --- | --- | --- | --- | --- | --- |
|  |  | **Pench** | **Melghat** | **Tadoba** | **Nagzira** | **Kanha** | **Nagarjunsagar** |
| **Into** | **Pench** | 0.822 |  |  |  |  |  |
|  | **Melghat** | 0.031 | 0.739 |  |  |  |  |
|  | **Tadoba** | 0.005 | 0.005 | 0.977 |  |  |  |
|  | **Nagzira** | 0.023 | 0.027 | 0.005 | 0.715 |  |  |
|  | **Kanha** | 0.098 | 0.047 | 0.005 | 0.023 | 0.840 |  |
|  | **Nagarjunsagar** | 0.018 | 0.032 | 0.004 | 0.024 | 0.029 | 0.903 |

Matrix shows the migration rates from the population in the horizontal row to the population in the vertical column
